# Enhanced DNA adduct formation by benzo[*a*]pyrene in human liver cells lacking cytochrome P450 oxidoreductase

**DOI:** 10.1016/j.mrgentox.2020.503162

**Published:** 2020-04

**Authors:** Lindsay Reed, Ian W.H. Jarvis, David H. Phillips, Volker M. Arlt

**Affiliations:** a Department of Analytical, Environmental and Forensic Sciences, MRC-PHE Centre for Environment and Health, King’s College London, London, SE1 9NH, United Kingdom; b NIHR Health Protection Research Unit in Health Impact of Environmental Hazards, King’s College London in Partnership With Public Health England and Imperial College London, London, SE1 9NH, United Kingdom

**Keywords:** Cytochrome P450, Cytochrome P450 oxidoreductase, Gene knockout, Metabolism, DNA adducts

## Abstract

Diet is a major source of human exposure to polycyclic aromatic hydrocarbons (PAHs), of which benzo[*a*]pyrene (BaP) is the most commonly studied and measured. BaP has been considered to exert its genotoxic effects after metabolic activation by cytochrome P450 (CYP) enzymes whose activity can be modulated by cytochrome P450 oxidoreductase (POR), the electron donor to CYP enzymes. Previous studies showed that BaP-DNA adduct formation was greater in the livers of Hepatic Reductase Null (HRN) mice, in which POR is deleted specifically in hepatocytes, than in wild-type (WT) mice. In the present study we used human hepatoma HepG2 cells carrying a knockout (KO) in the *POR* gene as a human *in vitro* model that can mimic the HRN mouse model. Treatment to BaP for up to 48 h caused similar cytotoxicity in POR KO and WT HepG2 cells. However, levels of BaP activation (*i.e.* BaP-7,8-dihydrodiol formation) were higher in POR KO HepG2 cells than in WT HepG2 cells after 48 h. This also resulted in substantially higher BaP-DNA adduct formation in POR KO HepG2 cells indicating that BaP metabolism is delayed in POR KO HepG2 cells thereby prolonging the effective exposure of cells to unmetabolized BaP. As was seen in the HRN mouse model, these results suggest that cytochrome *b_5_*, another component of the mixed-function oxidase system, which can also serve as electron donor to CYP enzymes along with NADH:cytochrome *b_5_* redutase, contributes to the bioactivation of BaP in POR KO HepG2 cells. Collectively, these findings indicate that CYPs play a more important role in BaP detoxication as opposed to activation.

## Highlights

• Cytochrome P450 oxidoreductase (POR) and cytochrome *b_5_* modulate CYP enzyme activity.
• CYP-mediated activation of benzo[*a*]pyrene (BaP) was studied in POR knockout (KO) cells.
• BaP-7,8-dihydro-diol levels were increased in POR KO cells after 48 h.
• BaP-DNA adduct formation was enhanced in POR KO cells after 48 h.
• Cytochrome *b_5_* seems to modulate CYP-mediated BaP bioactivation in POR KO cells.

## 1 Introduction

**Fig. 1:**
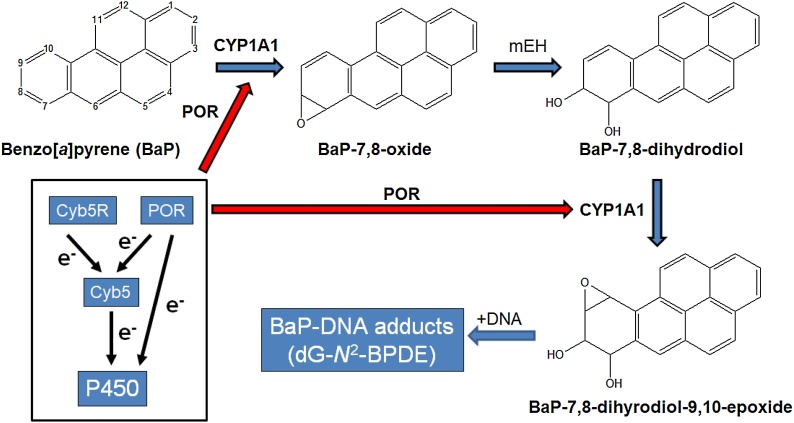
Relevant pathway of BaP biotransformation and BaP-DNA adduct formation catalysed by CYP1A1 and mEH. The three-stage pathway leads to the formation of the ultimately reactive species BPDE that binds to guanine to form the dG-*N*^2^-BPDE adduct. The box shows the roles of POR, Cyb5R and Cyb5 as electron donors to P450 enzymes. The red arrows indicate the step where POR KO can impact on the CYP1A1-mediated bioactivation of BaP.

Humans are constantly and unavoidably exposed to a myriad of environmental chemical substances. Polycyclic aromatic hydrocarbons (PAHs) are ubiquitous environmental contaminants formed during incomplete combustion or pyrolysis of organic matter [1]. Diet is a major source of human PAHs exposure with highest PAH levels in foods reported in cooked, smoked and charcoal grilled meat [2]. One such PAH is benzo[*a*]pyrene (BaP; Fig. 1) [3,4] that requires metabolic activation before reacting with DNA to exert its genotoxic effects [5]. Based on experiments with cell-free systems *in vitro*, cytochrome P450s (CYPs) enzymes have an important role in the bioactivation of many PAHs [6], with mainly CYP1A1 and CYP1B1 oxidising BaP initially to BaP-7,8-epoxide (Fig. 1), which is then converted to BaP-7,8-dihydrodiol (Fig. 1) by microsomal epoxide hydrolase (mEH) [5]. Further activation of BaP-7,8-dihydrodiol by CYP1A1 leads to BaP-7,8-dihydrodiol-9,10-epoxide (BPDE; Fig. 1) which is the ultimate reactive intermediate capable of reacting with DNA to form preferentially pre-mutagenic guanine adducts [*i.e.* 10-(deoxyguanosin-*N*^2^-yl)-7,8,9-trihydroxy-7,8,9-tetrahydro-BaP; dG-*N*^2^-BPDE] [7,8].

CYP enzymes, including CYP1A1, are components of a mixed-function oxidase system that also contains other enzymes, including the multidomain flavoprotein NADPH:cytochrome P450 oxidoreductase (POR) and cytochrome *b_5_* (Cyb5) accompanied by NADH:cytochrome *b_5_* reductase (Cyb5R) that serve as electron donors to CYP enzymes. We previously used the Hepatic P450 Reductase Null (HRN) and Hepatic Cytochrome *b_5_*/P450 Reductase Null (HBRN) mouse models [9] to study the impact of POR and Cyb5 on the CYP-catalysed bioactivation of BaP. Strikingly, BaP-induced DNA adduct formation was substantially increased in the livers of HRN mice compared to wild-type (WT) mice [7,10] whereas in HBRN mice BaP-DNA adduct levels were significantly lower than in HRN mice, whilst no significant difference was observed compared to WT mice [11]. The increased levels of BaP-DNA adducts in the livers in HRN mice were in accordance with other studies conducted in *Cyp1a1(‒/‒)* mice treated with BaP (reviewed in [12]) revealing an apparent paradox, whereby hepatic CYP enzymes appear to be more important for BaP detoxification *in vivo*, despite being involved in its metabolic activation *in vitro* (reviewed in [13]). Interestingly, we recently showed that the results observed in HRN mice could be recapitulated in cultured mouse hepatoma Hepa1c1c7 cells that carried a gene knockout in *POR* (*i.e.* POR KO Hepa1c1c7 cells) [14], in which, BaP-DNA adduct levels were approximately 10-fold higher in POR KO Hepa1c1c7 cells than in WT Hepa1c1c7 cells following exposure to BaP. In order to investigate whether the phenomenon observed in POR KO Hepa1c1c7 cells extends to human cells, we have now conducted a study in the human hepatoma cell line HepG2.

HepG2 cells are frequently used as *in vitro* alternative to primary human hepatocytes [15]. Although they have reduced levels of some metabolic activities than primary human hepatocytes they have been shown to undergo induction of phase I and phase II enzymes involved in BaP metabolism and BaP-induced DNA adduct formation [16]. Generally, HepG2 cells have been described as a sensitive model for identifying and quantifying DNA-damaging properties of environmental and dietary agents [17]. Several studies have shown that BaP-DNA adduct formation increases in HepG2 cells in a concentration-dependent manner [[18], [19], [20]]. BaP treatment of HepG2 cells also led to altered expression of genes involved in xenobiotic metabolism, cell cycle regulation, apoptosis/anti-apoptosis, chromatin assembly and oxidative stress response [18]. The overall response to BaP consisted of up-regulation of tumour suppressor genes and down-regulation of oncogenes promoting cell cycle arrest and apoptosis. Anti-apoptotic signalling that may increase cell survival and promote tumourigenesis was also evident [18].

CYP activity has also been shown to play an important role in the metabolism of BaP in HepG2 cells: when cells were pretreated with 2,3,7,8-tetrachlorodibenzo-*p*-dioxin (TCDD) and subsequently exposed to BaP, BaP-DNA-adduct formation as well as hypoxanthine-guanine phosphribosyltransferase (HPRT) mutation frequency were reduced through the induction of CYP1A1. These effects were shown to be even more pronounced in cells exposed to BPDE after TCDD pretreatment [21], suggesting that increased levels of CYP1A1 are protective against BaP-induced DNA damage.

The aim of the present study was to use cultured human cells that lack POR protein expression to investigate whether this human *in vitro* model mimics the results observed in the HRN mouse model *in vivo* [7,10,11] and the POR KO Hepa1c1c7 mouse model *in vitro* [14], which both formed higher levels of DNA adducts after BaP exposure than WT mice and cells, respectively. For this approach, a POR KO HepG2 cell line was used alongside WT HepG2 cells. The response of the cells to BaP exposure was investigated by analysing cytotoxicity, expression of xenobiotic-metabolising enzymes (XMEs), BaP metabolite formation and BaP-induced DNA damage (*i.e.* BaP-DNA adduct formation).

## 2 Materials and methods

### 2.1 Carcinogen

Benzo[*a*]pyrene (BaP, CAS number 50-32-8; purity ≥96 %) was obtained from Sigma Aldrich (UK).

### 2.2 Cell culture and BaP treatment

Human hepatocellular carcinoma HepG2 cells were originally obtained from the American Type Culture Collection (ATCC). POR KO HepG2 cells (#HG2-101) were obtained from HeraBioLabs (Lexington, KY, USA). Cells were cultured in 75-cm^2^ flasks (Thermo Scientific) using MEM (Life Technologies) with 10 % FBS (Invitrogen), 1% 100 mM sodium pyruvate, 1% 100X non-essential amino acids and 1% penicillin streptomycin (Life Technologies) using a Heraeus HERAcell™ humidified incubator which was set at 37 °C, 5% CO_2_ and 95 % air.

Cells were seeded into 6-well plates at approximately 0.48 × 10^5^ cells per well or 15 × 10^5^ cells per 75-cm^2^ flask and treated 48 h post seeding. Based on previous experiments [18], WT and POR KO HepG2 cells were treated with up to 10 μM BaP (0.1 % dimethyl sulfoxide [DMSO] final) for 24 or 48 h. BaP was diluted in complete growth medium prior to treatment. Cells grown in medium containing 0.1 % DMSO alone served as control.

### 2.3 Cell viability

Cell survival after BaP treatment was determined using the crystal violet staining assay as described previously [14].

### 2.4 Western blot analysis

Preparation of whole cell lysates, separation of proteins by sodium dodecyl sulphate–polyacrylamide gel electrophoresis using 4–12 % Bis-Tris gradient gels and Western blotting was performed as previously reported [14]. The following primary antibodies were used, all diluted in 3% TBST milk: anti-POR 1:1000 (ab39995; Abcam), anti-CYP1A1 1:1000 (sc-20772 [H-70]; Santa Cruz Biotechnology), anti-Cyb5 1:500 (sc-33174 [H-114]; Santa Cruz Biotechnology), anti-Cyb5R 1:1000 (ABIN453978; Antibodies-online.com), and glyceraldehyde phosphate dehydrogenase (GAPDH) 1:2500 (MAB374; Chemicon). Visualisation of bands was accomplished by chemiluminescence detection using ECLTM Western Blotting reagents (GE Healthcare, Amersham) according to the manufacturer’s instructions and exposing the membranes to film.

### 2.5 HPLC analysis

BaP metabolite formation in the culture medium was determined by HPLC as reported [14,22]. Using authentic standards, (±)-*trans*-7,8-dihydroxy-7,8-dihydro-BaP (BaP-7,8-dihydrodiol) and (±)-*r*-7,*t*-8,*t*-9,*c*-10-tetrahydroxy-7,8,9,10-tetrahydro-BaP (BaP-tetrol-I-1) were the two BaP metabolites identified [23].

### 2.6 ^32^P-postlabelling assay

The nuclease P1 enrichment version of the thin-layer chromatography (TLC) ^32^P-postlabelling assay was used to detect DNA adducts as reported [14,24]. After chromatography, TLC sheets were scanned using phosphor-imaging technology (Amersham™ Typhoon IP; GE Healthcare). Adduct levels were calculated as relative adduct labelling (RAL) values, which represent the ratio of count rates of adducted nucleotides (adducts) over count rates of total (adducted plus normal) nucleotides in the DNA sample analysed. A deoxyadenosine-3′-monophosphate (dAp) standard was labelled in each experiment to estimate the count rates of total nucleotides. Results are expressed as DNA adducts/10^8^ nucleotides.

### 2.7 Statistical analysis

All statistical analyses were performed with the Prism GraphPad Software (Version 8.0.0) and *P* < 0.05 was considered significant. Area under the curve (AUC) for cell viability were compared by two-way Anova with Tukey’s multiple comparison test.

## 3 Results

### 3.1 BaP-induced cytotoxicity

**Fig. 2:**
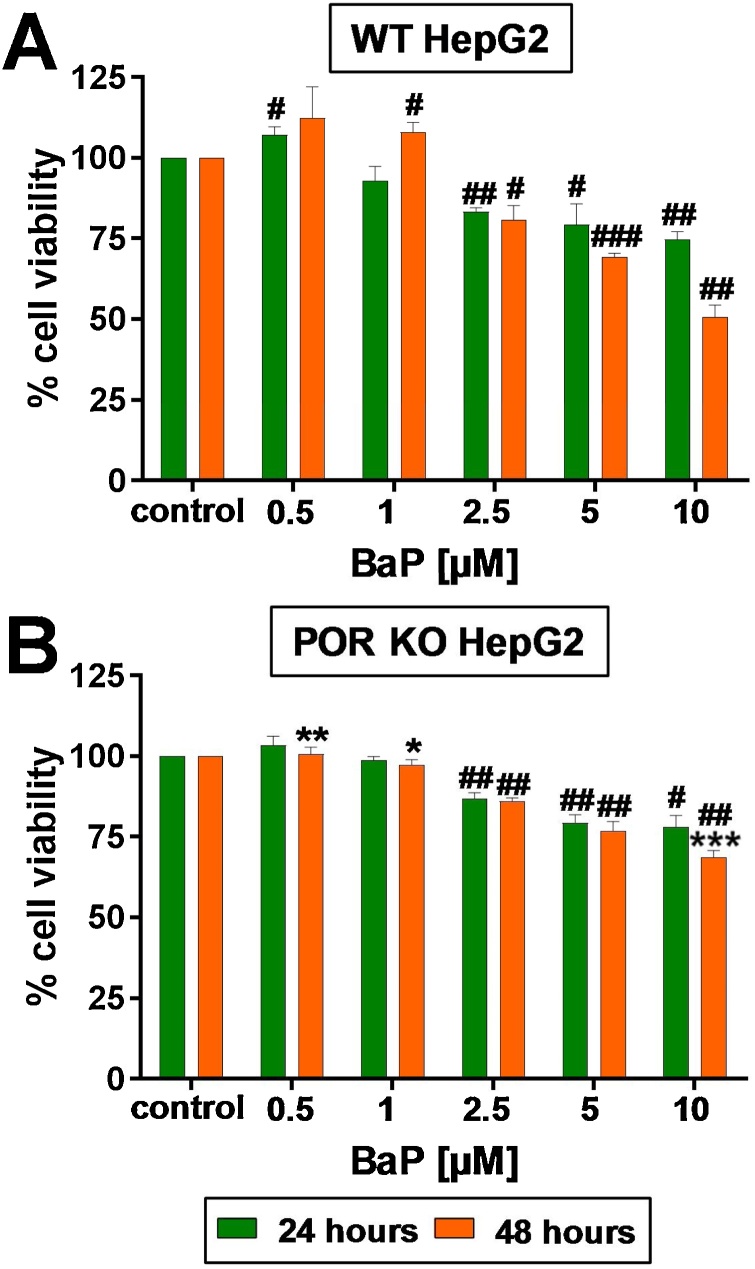
Cell viability (% control [untreated]) of WT (A), KO POR (B) HepG2 cells after treatment with a range of BaP concentrations over a 24- and 48 -h period. Values are given as mean ± SD (*n* = 3); each assay was repeated in separate experiments. To evaluate the effects of BaP treatment on cell viability in each cell line, the viability data were normalised to 1.0, data then log2 transformed and analysed using a single sample *t*-test with Bonferroni correction against the population control mean of 0 (^#^ = compared to control [*i.e.* untreated], ^#^*P* ≤ 0.05, ^##^*P* ≤ 0.01, ^###^*P* ≤ 0.001). To evaluate the effects of BaP treatment on cell viability between both cell lines, statistical analysis was performed by two-way Anova with Tukey’s multiple comparison test (* = compared to WT, * *P* ≤ 0.05; ** *P* ≤ 0.01; *** *P* ≤ 0.001).

WT and POR KO HepG2 cells were treated with a range of BaP concentrations (0–10 μM) over a 24- or 48 -h period (Fig. 2). In both cell lines cell viability decreased in a concentration-dependent manner. POR KO HepG2 cells showed a lesser degree of difference between 24- and 48 -h exposure times at the different BaP concentrations tested (Fig. 2B) when compared with WT HepG2 cells (Fig. 2A). POR KO HepG2 cells showed significantly less cytotoxicity than WT HepG2 cells after 48 h exposure at the highest concentration of 10 μM (Fig. 2B). A comparison of the AUC confirmed that the response of the POR KO HepG2 cells at 48 h is significantly different (*P* < 0.01) relative to the WT HepG2 cells after BaP exposure. Based on the cytotoxicity data in the WT HepG2 cells (Fig. 2A), 2.5 and 5 μM BaP were selected for further experiments, which led to ∼70 % cell viability over 24 or 48 h.

### 3.2 Protein expression of XMEs

**Fig. 3:**
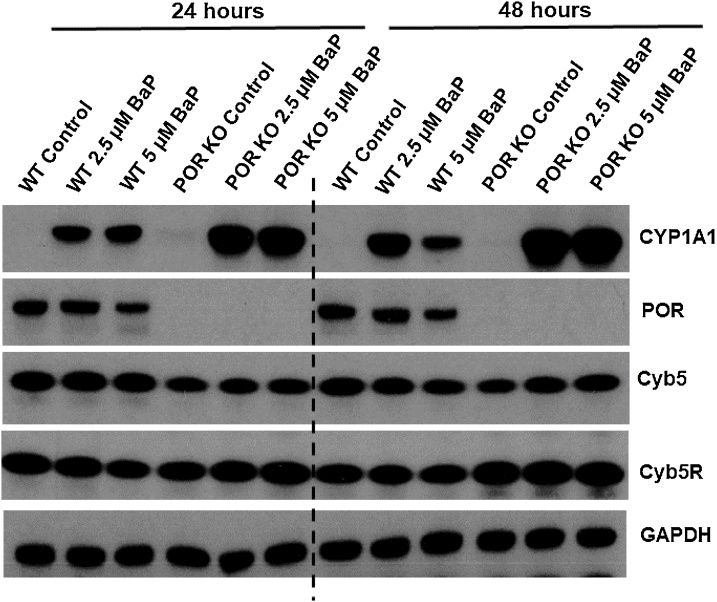
Western blot analysis of CYP1A1, POR, Cyb5 and Cyb5R in untreated (control) and BaP-treated (2.5 or 5 μM for 24 or 48 h) WT and KO POR HepG2 cells. Representative images of the Western blotting are shown, and at least duplicate analysis was performed from independent experiments. GAPDH protein expression was used as a loading control.

WT and POR KO HepG2 cells were treated with 2.5 or 5 μM BaP for 24 or 48 h and expression of POR, Cyb5, Cyb5R and CYP1A1 were determined by Western blotting (Fig. 3). As expected, POR was expressed in WT HepG2 cells, but no expression was observed in the POR KO HepG2 cells. Cyb5 and Cyb5R were expressed in both cell lines. Treatment with BaP did not majorly alter the levels of POR, Cyb5 and Cyb5R expression relative to untreated controls. However, it may appear that in WT HepG2 cells POR expression is lower at the highest BaP concentration (5 μM) relative to controls, at both timepoints. In contrast, CYP1A1 was induced in both cell lines by BaP treatment; and the extent of CYP1A1 induction by BaP was substantially greater in POR KO HepG2 cells than in WT HepG2 cells.

### 3.3 Analysis of BaP metabolites

**Fig. 4:**
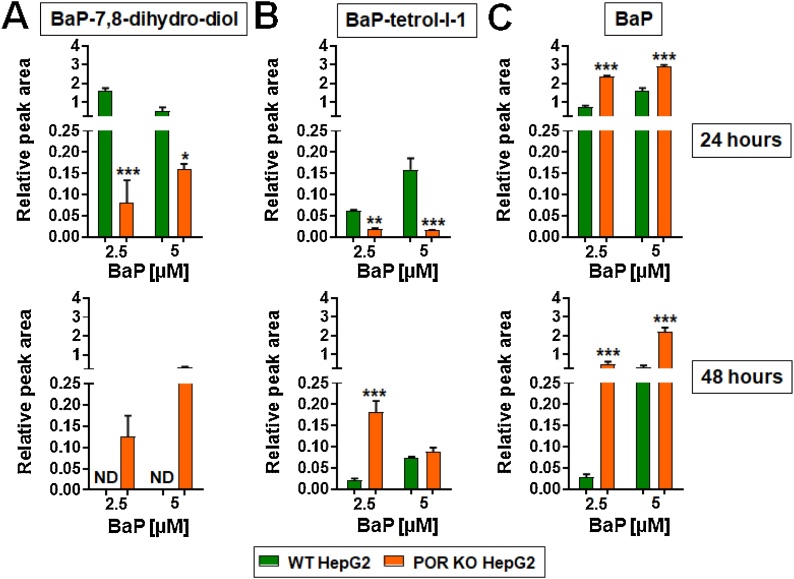
Formation of BaP-7,8-dihydrodiol (A), BaP-tetrol-I-1 (B) and BaP (C) in WT and POR KO HepG2 cells after treatment with 2.5 or 5 μM BaP for 24 (*upper panels*) or 48 (*lower panels*) hours. Values are given as mean ± SD (*n* = 3); each assay was repeated in separate experiments. Statistical analysis was performed by one-way Anova with Tukey’s multiple comparison test (* = compared to WT, * *P* ≤ 0.05 ** *P* ≤ 0.01 *** *P* ≤ 0.001).

WT and POR KO HepG2 cells were treated with 2.5 and 5 μM BaP for 24 or 48 h before the culture medium was removed and extracted. Extracts were then analysed by HPLC to determine the BaP metabolite profile (Fig. 4). BaP-7,8-dihydrodiol, the precursor to BPDE, and BaP-tetrol-I-1, one hydrolysis product of BPDE, were the two metabolites detected along with unmetabolised BaP. BaP-7,8-dihydrodiol (Fig. 4A) and BaP-tetrol-I-1 (Fig. 4B) were both at significantly higher levels in culture medium from WT HepG2 cells than from POR KO HepG2 cells after exposure for 24 h at both 2.5 or 5 μM BaP. After exposure to 2.5 μM BaP, but not 5 μM, for 48 h, BaP-tetrol-I-1 formation was significantly higher with POR KO HepG2 cells (Fig. 4B). After exposure to either concentration of BaP for 48 h, BaP-7,8-dihydrodiol was detected in the medium of POR KO HepG2 cells, but not of WT HepG2 cells (Fig. 4A). Thus, the levels of BaP-metabolites declined in the WT HepG2 cells from 24 to 48 h after treatment with either 2.5 or 5 μM BaP, while they increased over the exposure time in the POR KO HepG2 cells (Fig. 4A & B). The level of unmetabolised BaP was significantly higher in the medium from POR KO HepG2 cell at both concentrations and timepoints (Fig. 4C). No BaP or BaP metabolites were detected in untreated cells (data not shown).

### 3.4 Analysis of DNA adduct formation

**Fig. 5:**
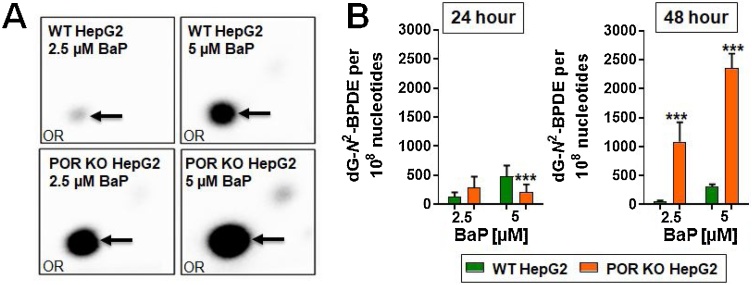
DNA adduct formation in WT and POR KO HepG2 cells after treatment with 2.5 or 5 μM BaP for 24 or 48 h. (A) Representative autoradiograms showing adduct profiles by TLC ^32^P-postlabelling in HepG2 cells exposed to BaP for 48 h. The origins (ORs) on the TLC plates, at the bottom left-hand corner, were cut off before imaging. The arrow indicates the dG-*N*^2^-BPDE adduct used for quantitation. (B) Quantitative TLC ^32^P-postlabelling analysis of dG-*N*^2^-BPDE in BaP-treated WT and POR KO HepG2 cells. Values are given as mean ± SD (*n* = 4); each DNA sample was collected in separate experiments and analysed in the same ^32^P-postlabelling run. Statistical analysis was performed by one-way Anova with Tukey’s multiple comparison test (* = compared to WT, *** *P* ≤ 0.001).

Analysis of DNA adducts by ^32^-P-postlabelling detected one major adduct spot in all BaP-treated cells (Fig. 5A), which has previously been identified as dG-*N*^2^-BPDE [7]. No DNA adducts were detected in untreated cells (data not shown).

After treatment with 2.5 μM BaP for 24 h differences in BaP-DNA adduct formation between WT and POR KO HepG2 cells were not significant; however, adduct formation was significantly lower in POR KO HepG2 cells than in WT HepG2 cells 24 h after treatment with 5 μM BaP (Fig. 5B). In contrast, adduct levels were significantly higher in POR KO HepG2 cells 48 h after treatment with 2.5 and 5 μM BaP (Fig. 5B). The levels of dG-*N*^2^-BPDE in POR KO HepG2 cells correlated with BaP-7,8-dihydrodiol formation after 24 and 48 h (compare Fig. 4).

## 4 Discussion

Previously we created mouse hepatoma Hepa1c1c7 cells lacking POR expression using CRISPR/Cas9 technology [14]. Treatment of WT and POR KO Hepa1c1c7 cells with BaP resulted in significantly higher BaP metabolite and BaP-DNA adduct formation in POR KO Hepa1c1c7 cells than in WT Hepa1c1c7 cells. Higher BaP-DNA adduct formation in these mouse liver cells that lack POR was in accordance with enhanced BaP-DNA adduct formation *in vivo* observed in the livers of BaP-treated HRN mice lacking POR [7,10,11]. In contrast, studies using microsomal fractions isolated from the livers of HRN mice [7,11] and reconstituted systems (*i.e.* presence and absence of purified POR) [25] showed the importance of POR in catalysing CYP-mediated BaP-DNA adduct formation *in vitro* in cell-free systems. Collectively these studies have revealed paradoxical results regarding the role of CYP enzymes in the activation or detoxication of BaP. In order to investigate whether the phenomenon observed in POR KO Hepa1c1c7 cells extends to human cells, we used POR KO HepG2 cells in the present study.

Western blotting confirmed the absence of POR protein in the POR KO HepG2 cells and its presence in WT HepG2 cells. Expression of other electron donor proteins (*i.e.* Cyb5, Cyb5R) did not majorly change in either cell line irrespective of BaP concentration or exposure time. This contrasts with results seen with HRN mice and POR KO Hepa1c1c7 cells, where Cyb5 expression was induced after BaP treatment [10,14]. CYP1A1 protein was not detectable in untreated WT and POR KO HepG2 cells under the experimental conditions used but was greatly induced by BaP. Expression of CYP1A1 was greater in POR KO HepG2 cells than in WT HepG2 cells, with higher expression of CYP1A1 in KO POR HepG2 cells after 48 h than at 24 h. Despite the absence of POR expression, POR KO HepG2 cells did not exhibit the same degree of resistance to BaP toxicity that was seen previously in POR KO Hepa1c1c7 cells over either a 24- or 48 -h period [14], which suggests that BaP is biotransformed into toxic metabolites at similar levels as in WT HepG2 cells. However, in fact BaP-7,8-dihydrodiol formation was significantly lower in POR KO HepG2 cells than in WT HepG2 cells after exposure for 24 h, while the opposite was found after 48 h. The higher levels of BaP-7,8-dihydrodiol in POR KO HepG2 cells after BaP exposure for 48 h correlated with results previously seen in POR KO Hepa1c1c7 cells [14]. The delayed BaP metabolism in POR KO HepG2 cells relative to WT HepG2 cells is also reflected by the massive increase in DNA adduct formation in POR KO HepG2 cells after 48 h, regardless of the concentration tested. The dG-*N*^2^-BPDE adduct is the same adduct as seen in the HRN mouse model and the accumulation of BaP-DNA adducts in POR KO HepG2 cells correlates with previous studies carried out with the HRN mouse [7,10] and POR KO Hepa1c1c7 cells [14].

The responses seen in the present study are clearly time-dependent, with POR KO HepG2 cells displaying the greatest levels of BaP metabolite and dG-*N*^2^-BPDE adduct formation 48 h after treatment, whereas in WT HepG2 cells this was observed after 24 h. BaP-DNA adduct formation has previously been assessed in WT HepG2 cells over a range of BaP concentrations (0.01–5 μM) and time points. Whilst concentration was found to affect levels of DNA adducts, there was no significant difference between the 6-, 24- and 48 -h time points for each concentration suggesting that BaP is rapidly metabolised in WT HepG2 cells [18]. Thus, the time-dependent differences in the POR KO HepG2 cells could be indicative of slower clearance of BaP compared to WT HepG2 cells. Slower metabolic clearance of BaP was observed in *Cyp1a1(‒/‒)* mice treated with BaP which resulted in greater BaP-DNA adduct formation in the livers of these mice [26].

Although higher BaP-DNA adduct levels in POR KO HepG2 cells 48 h after BaP exposure correlates with findings previously seen in BaP-exposed POR KO Hepa1c1c7 cells after 24 h, the kinetics of DNA adduct formation are different in mouse and human cells. It can be speculated that the metabolic competence, *i.e.* the expression of BaP metabolising enzymes, of HepG2 and Hepa1c1c7 cells is different. For example, previous results have shown that human breast carcinoma MCF-7 cells are more efficient than HepG2 cells in mediating BaP-DNA adduct formation [18]. After 24-h exposure, MCF-7 cells formed around 100 adducts/10^8^ nucleotides when treated with a concentration of 0.25 μM BaP whereas a 10-fold higher BaP concentration (2.5 μM) was required in HepG2 cells to achieve the same DNA adduct level. Furthermore, whereas BaP-DNA adduct formation was clearly time-dependent at all concentrations when testing BaP treatment for 6 and 24 h in MCF-7 cells, no such time-dependent effect was seen in HepG2 cells (*i.e.* adduct levels were always quite similar after 6- and 24 -h treatment). Thus, it is clear that MCF-7 cells, like Hepa1c1c7 cells, are more sensitive than HepG2 cells, which impacts on the kinetics of BaP-DNA adduct formation in these cells. Comparing the present results in POR KO HepG2 and WT HepG2 cells indicates that the loss of POR leads to further alterations to the kinetic, providing a possible explanation for why the lack of POR impacts at different timepoints in POR KO HepG2 and POR KO Hepa1c1c7 cells.

Loss of POR in HRN and HBRN mice resulted in steatotic livers [27,28] that could have stored BaP [7]. Lipid content of the POR KO HepG2 cells was not assessed; however, if a similar effect on lipid metabolism was to occur in POR KO HepG2 cells it could alter BaP bioavailability and metabolism, including detoxication. Levels of unmetabolised BaP in the cell culture media of POR KO HepG2 cells were significantly higher than with WT HepG2 cells regardless of concentration or time, however whether this is because of reduced cellular uptake of BaP due to slower metabolism is unknown. The levels of BaP sequestered in HepG2 cells were not assessed but a combination of slower metabolic clearance of BaP and higher levels of sequestered BaP could explain the accumulation of dG-*N*^2^-BPDE adducts in POR KO HepG2 cells. The accumulation of dG-*N*^2^-BPDE adducts in HRN mice [10] and POR KO Hepa1c1c7 cells [14] was associated with increased levels of Cyb5 protein expression, however no difference in Cyb5 expression was observed regardless of concentration or time between POR KO and WT HepG2 cells despite the accumulation of dG-*N*^2^-BPDE adduct formation in the POR KO HepG2 cells. Still, it is probable that Cyb5 compensates for the lack of POR and contributes to increased P450-mediated activation of BaP as seen in reconstituted (cell-free) systems [29].

Although HepG2 cells have inducible CYP1A1 [18], they lack pregnane X receptor (PXR) expression, which is a low affinity nuclear receptor that transcriptionally regulates many genes associated with xenobiotic metabolism. It plays an important role in detoxication, to the extent that PXR is considered to be a xenosensor due to the broad range of xenobiotic substrates including pharmaceuticals, dietary nutrients and environmental contaminants [30]. In order to assess the role of PXR and the subsequent upregulation of XMEs that metabolise BaP, previous studies have used a PXR-transfected HepG2 cell line [19]. Formation of BaP-DNA adducts in PXR-overexpressing HepG2 cells was significantly lower than in WT HepG2 cells, suggesting that the presence of PXR reduces the formation of reactive BaP intermediates (*i.e.* BPDE). BaP-DNA adduct formation was also significantly reduced in PXR-overexpressing HepG2 cells that had been pretreated with rifampicin, a CYP3A4 inducer, indicating the importance of CYPs for detoxication in this model [19]. After exposure to BaP, mRNA levels of *CYP1A2* and *GSTP1* (glutathione S-transferase P1) were notably higher in the PXR-overexpressing cells HepG2 than in WT HepG2 cells. Levels of *CYP1A1* mRNA were also increased in both cell lines after BaP treatment, however this effect was attenuated in PXR-transfected cells [19]. This correlates with results observed in *Cyp1a1(‒/‒)* mice where absence of Cyp1a1 activity led to increased levels of BaP-DNA adduct formation [26,31]. The results from the present study also indicate that CYP enzymes play a more important role in the detoxication of BaP as opposed to bioactivation.

Other XMEs have been implicated in the activation of BaP including aldo-keto reductases (AKRs) [32] and cyclooxygenases (COXs) [33]. HepG2 cells have been shown to have significantly increased levels of AKR gene expression after treatment with BaP [18,35]. AKRs have been shown to catalyse the oxidation of BaP-7,8-dihydrodiol to form a ketol, which tautomerises to form the air-sensitive BaP-7,8-catechol [32]. This catechol undergoes one-electron oxidation in air to form an o-semiquinone anion radical and a subsequent one-electron oxidation in air to form the fully oxidised BaP-7,8-dione (o-quinone) that leads to DNA base oxidation (*e.g.* 8-oxo-dGuo) [32]. Studies with AKR1A1-transfected human bronchoalveolar cells showed that AKRs and CYP enzymes compete in the activation of BaP-7,8-dihydrodiol [35]. A later study utilising AKR1A1-transfected cells found that the same major adduct, dG-*N*^2^-BPDE, was formed as in CYP1A1/1B1-induced cells when treated with BaP-7,8-dihydrodiol. However, AKR1A1-transfected cells exhibited a 3-h lag phase before significant BaP-DNA adduct formation (*i.e.* dG-*N*^2^-BPDE) was detected [36]. In POR KO HepG2 cells therefore, AKRs could potentially contribute to BaP-DNA adduct formation. On the other hand, previous findings showed that WT HepG2 cells exhibited the highest levels of DNA adduct formation compared to AKR1A1-transfected or CYP1A1/1B1-induced HepG2 cells, indicating that ARK1A1 and CYP1A1/CYP1B1 may be protective against BPDE-mediated DNA damage [36]. Whilst COX1/2 has previously been implicated in the activation of BaP, no significant changes in gene expression were seen in HepG2 cells treated with BaP [18,34]. Further investigation of these enzymes and the effect of BaP on their expression in the POR KO HepG2 cells as opposed to WT HepG2 cells could offer further insight into enzymes involved in the bioactivation of BaP. This could then help to identify potential P450-independent BaP activation pathways in the POR KO HepG2 cells and be the focus of future studies.

## 5 Conclusions

In the present study we showed that levels of BaP activation (*i.e.* BaP-7,8-dihydrodiol formation) were higher in POR KO HepG2 cells than in WT HepG2 cells after 48 h, which resulted in substantially higher BaP-DNA adduct formation (*i.e.* dG-*N*^2^-BPDE) in POR KO HepG2 cells. It appears that in POR KO HepG2 cells BaP metabolism is delayed relative to WT HepG2 cells, which prolongs the effective exposure of the cells to unmetabolised BaP, ultimately producing greater amounts of genotoxic BaP intermediates (*i.e.* BPDE). These findings correlate with those previously seen in HRN mice and POR KO Hepa1c1c7 cells, where the absence of POR expression resulted in enhanced BaP-DNA adduct formation. Collectively, these results indicate that CYPs play a more important role in the detoxication of BaP than in its activation. Future studies will aim to additionally knock out Cyb5 in POR KO HepG2 cells in order to specifically assess the contribution of Cyb5 in CYP-mediated BaP bioactivation.

## Funding information

Lindsay Reed was supported by a King’s College London Health Faculty PhD Studentship funded by the Medical Research Council (Grant 1524896). Work at King’s College London was further supported by the National Institute for Health Research Health Protection Research Unit (NIHR HPRU) in Health Impact of Environmental Hazards at King’s College London in partnership with Public Health England (PHE) and Imperial College London.

## Declaration of Competing Interest

The authors declare that there are no conflicts of interest.
